# A Novel Ultrasensitive In Situ Hybridization Approach to Detect Short Sequences and Splice Variants with Cellular Resolution

**DOI:** 10.1007/s12035-017-0834-6

**Published:** 2017-12-20

**Authors:** Larissa Erben, Ming-Xiao He, Annelies Laeremans, Emily Park, Andres Buonanno

**Affiliations:** 1 Section on Molecular Neurobiology, Eunice Kennedy Shriver National Institute of Child Health and Human Development, National Institutes of Health, Porter Neuroscience Research Center, Bldg. 35, Room 2C-1000, Bethesda, MD 20892 USA; 2 Institute of Molecular Psychiatry, University Bonn, 53127 Bonn, Germany; 3 Advanced Cell Diagnostics, Newark, CA 94560 USA

**Keywords:** Schizophrenia, ErbB4, Neuregulin, Alternative splicing, Oligodendrocytes, RNA expression, Transcriptome, BaseScope

## Abstract

Investigating the expression of RNAs that differ by short or single nucleotide sequences at a single-cell level in tissue has been limited by the sensitivity and specificity of in situ hybridization (ISH) techniques. Detection of short isoform-specific sequences requires RNA isolation for PCR analysis—an approach that loses the regional and cell-type-specific distribution of isoforms. Having the capability to distinguish the differential expression of RNA variants in tissue is critical because alterations in mRNA splicing and editing, as well as coding single nucleotide polymorphisms, have been associated with numerous cancers, neurological and psychiatric disorders. Here we introduce a novel highly sensitive single-probe colorimetric/fluorescent ISH approach that targets short exon/exon RNA splice junctions using single-pair oligonucleotide probes (~ 50 bp). We use this approach to investigate, with single-cell resolution, the expression of four transcripts encoding the neuregulin (NRG) receptor ErbB4 that differ by alternative splicing of exons encoding two juxtamembrane (JMa/JMb) and two cytoplasmic (CYT-1/CYT-2) domains that alter receptor stability and signaling modes, respectively. By comparing ErbB4 hybridization on sections from wild-type and ErbB4 knockout mice (missing exon 2), we initially demonstrate that single-pair probes provide the sensitivity and specificity to visualize and quantify the differential expression of ErbB4 isoforms. Using cell-type-specific GFP reporter mice, we go on to demonstrate that expression of ErbB4 isoforms differs between neurons and oligodendrocytes, and that this differential expression of ErbB4 isoforms is evolutionarily conserved to humans. This single-pair probe ISH approach, known as BaseScope, could serve as an invaluable diagnostic tool to detect alternative spliced isoforms, and potentially single base polymorphisms, associated with disease.

**Electronic supplementary material:**

The online version of this article (10.1007/s12035-017-0834-6) contains supplementary material, which is available to authorized users.

## Introduction

Alternative mRNA splicing increases the functional complexity of the genome, with > 90% of all human multi-exon genes being differentially spliced [1]. In the central nervous system (CNS), alternative splicing is tightly regulated in a spatio-temporal manner, as well as by neuronal activity [2–4]. Different mRNA isoforms encode for ion channels, neurotransmitter receptors, adhesion molecules, and signaling proteins with distinct functional properties [5–8]. Splicing abnormalities are observed in different cancers and neurological diseases [9, 10], but are particularly abundant in psychiatric disorders, such as affective and addictive disorders, schizophrenia (Scz) and autism spectrum disorders [11]. In the postmortem brain of Scz patients, splice variant expression of many at-risk alleles is altered [12]; including those that encode: trophic factors [13–19], neuronal migration and adhesion proteins [20, 21], structural components of myelin and synapses [22, 23] and isoforms associated with dopaminergic, GABAergic and glutamatergic neurotransmission and signaling [24–28].

**Fig. 1:**
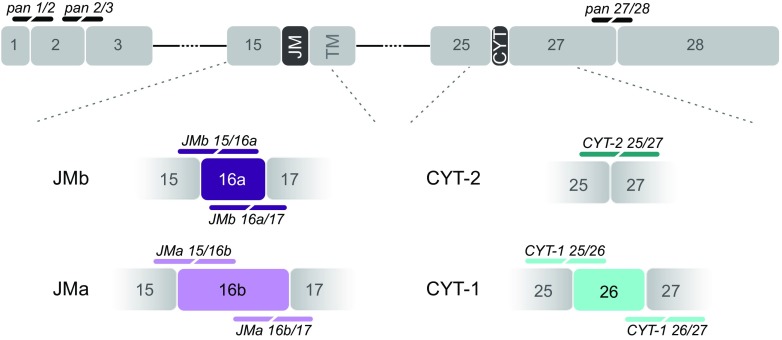
Scheme summarizing ErbB4 isoforms and single-pair probe design. ErbB4 isoforms are generated by alternative splicing of exons encoding the extracellular juxtamembrane domain, resulting in mutually exclusive JMa (exon 16b, *light purple*, 75 bp) or JMb (exon 16a, *dark purple*, 45 bp) isoforms, and by inclusion or exclusion of exon 26 encoding a region of the cytoplasmic domain giving rise to CYT-1 (*light cyan*, 48 bp) and CYT-2 (*dark cyan*) isoforms, respectively. Single-pair probes targeting all ErbB4 isoforms (pan 1/2, pan 2/3 and pan 27/28) are illustrated in black, whereas isoform-specific single-pair probes targeting splice junctions are color-matched with their respective isoforms. JM: juxtamembrane region; TM: transmembrane domain; CYT: cytoplasmic region

The NRG/ErbB4 signaling pathway, which is reported to be associated with a risk for Scz [29–32], and its endophenotypes [33], regulates neuronal differentiation, migration and plasticity in the CNS [34–36]. Alternative splicing of two exons encoding the extracellular juxtamembrane (JM) domain JMa (75 bp exon) or JMb (45 bp exon), and the inclusion or exclusion of a 48 bp exon in the cytoplasmic (CYT) domain, generates four ErbB4 receptor isoforms: JMa/CYT-1, JMa/CYT-2, JMb/CYT-1 and JMb/CYT-2 (Fig. 1; [37, 38]). ErbB4 transcript levels comprising JMa and CYT-1 exons are increased in the dorsolateral prefrontal cortex (DLPFC) of Scz subjects [29, 39–41], and single nucleotide polymorphisms in ERBB4 correlate with changes in receptor isoform expression and risk for Scz [29, 40, 41].

The four ErbB4 isoforms differ functionally. JMa-containing ErbB4 isoforms, but not JMb variants, are susceptible to extracellular metalloprotease-mediated cleavage followed by gamma-secretase intramembranous cleavage that releases a transcriptionally active intracellular domain (ICD) to regulate gene expression [38, 42–45]. CYT-1-containing isoforms encode a site for phosphatidyl inositol 3-kinase recruitment that increases the downstream signaling capacities of CYT-1 variants [37, 43].

Because of the different functions imparted by distinct splice variants, in this case ErbB4, it is critically important to identify the cells that express distinct isoforms. Whereas quantitative real-time PCR (qRT-PCR) and RNA sequencing (RNAseq) can be designed to detect specific RNA splice variants with high sensitivity in different brain regions, these methodologies require the disruption of dissected tissue to isolate RNA. The technical requirements of RNA isolation come at the expense of losing in vivo cell-type-specific resolution of splice variant expression. Traditionally, in situ hybridization (ISH) using radioactively- and fluorescently-labeled complementary RNA probes have provided the sensitivity to detect abundant transcripts at cellular level, but fail to unambiguously identify cells expressing rare splice variants. Recent advances in ISH using multiple non-radioisotropic oligonucleotide probe pairs targeting a single transcript, combined with chemical signal amplification [46, 47], enable specific and sensitive co-detection of rare transcripts (known as “multiplexing”; [48]). However, the optimal target lengths of these probes (> 300 bp) exceed the size of most alternative spliced variants. Due to these limitations, in the present study we implement a novel ISH approach based on an ultrasensitive amplification chemistry that allows the specific detection of mRNA exon junctions by a single pair of 18–25 bp anti-sense oligonucleotide probes targeting adjacent mRNA sequences; hereafter denoted as “single-pair probe”.

## Materials and Methods

For further details see Supplemental Information.

### Animals and Human Brain Samples

Homozygous ErbB4 knock-out (KO) mice lacking exon 2 [49] will be hereafter designated as ErbB4-Δ2 KO mice. CNP-GFP [50], NG2-GFP [51] and wild-type (WT) C57BL/6J mice were obtained from the Jackson Laboratory. GAD67-GFP mice [52], were a kind gift from Yuchio Yanagawa (Gunma University, Japan). All procedures were approved by the NIH Animal Care and Use Committee. Ground frozen human brain samples from four male adult control individuals were obtained from the Human Brain Collection Core (National Institute of Mental Health, NIMH).

### ISH

The novel single-pair probe ISH approach used here (BaseScope, Advanced Cell Diagnostics, Newark, CA) is based on the well-established multiplex fluorescent ISH RNAscope® (Advanced Cell Diagnostics [47]). The high specificity and sensitivity of both ISH technologies are reached by a unique probe design using ‘ZZ’ probe pairs and signal amplification, respectively. Advances in signal amplification over RNAscope® allow for the use of a single-pair probe in the BaseScope assay, consisting of a pair of 18–25 bp oligonucleotide sequences. To detect exon junctions, one oligonucleotide probe hybridizes to target sequences across the exon junction and the other probe to an immediately adjacent region. Targeted sequences of customized junction-specific ErbB4 ISH probes are listed in Table 1 and schematically illustrated in Fig. 1. RNAscope® probes were ErbB4 (Mm-ErbB4; Cat No. 318721), GAD-2 (Mm-GAD2-C2; Cat No. 415071-C2) and MAG (Mm-MAG-C3; Cat No. 446451-C3). Briefly, both RNAscope® and BaseScope ISH assays were performed on 8 μm-thick formalin-fixed paraffin-embedded sections of 10-week-old adult mice; prepared as described by [53]. Briefly, sections were deparaffinized in xylene, endogenous peroxidase activity was blocked by H_2_O_2_ treatment (10 min at RT) and sections were permeabilized by antigen retrieval (15 min at 100 °C) and a protease mixture (30 min at 40 °C). Probes were bound by incubation for 2 h at 40 °C, chemically amplified, and then labeled by fluorophores (multiplex ISH) or alkaline phosphatase conversion of FastRED dye (single-pair probe ISH).

**Table 1:** Exon junction-specific single-pair probes for the detection of distinct ErbB4 isoforms

Probe name	Exon junction	Specificity	Target sequence (5′ → 3′)
JMa* 15/16b	E15/E16b	JMa	CCAGGG/GTGTAACGGTCCCACTAGTCATGACTGCATTTACTACCC
JMa* 16b/17	E16b/E17	JMa	GGACGGGCCATTCCACTTTACCACAACACGCTAG/AACTCCAC
JMb* 15/16a	E15/E16a	JMb	CCCAGGG/GTGCATAGGTTCAAGCATTGAAGACTGCATCGGC
JMb* 16a/17	E16a/E17	JMb	GTTCAAGCATTGAAGACTGCATCGGCCTGACGGATAG/AACTCCAC
CYT-1 25/26	E25/E26	CYT-1	CATCTACACATCCAGAACAAGAATTGACTCCAATAGG/AGTGAAATTGGAC
CYT-1 26/27	E26/E27	CYT-1	CCATGTCGGGA/AATCAGTTTGTGTACCAAGATGGGGGCTTT
CYT-2 25/27	E25/E27	CYT-2	CCATCTACACATCCAGAACAAGAATTGACTCCAATAGG/AATCAGTTTGT
pan 1/2	E1/E2	All isoforms	TCTCAGTCAG/TGTGCGCAGGAACAGAGAACAAACTGAGCTCTCTCT
pan 2/3	E2/E3	All isoforms	GAGCACAACCGGGACCTCTCCTTCCTGCGG/TCTATCCGAG
pan 27/28	E27/E28	All isoforms	GCATGACAAGCCCAAACAAG/AATATCTGAATCCTGTGGAAGAGAACC

Name of ErbB4 single-pair probes correspond to the number of the targeted exon/exon junctions. All target sequences correspond to sense strand and exon junctions are indicated by the dash. *Juxtamembrane exons JMa and JMb are numbered for convenience as exon 16b and 16a, respectively, which correspond to exon 16 and 15b in [62]

### Immunostainings

Post hoc immunohistochemistry immediately following ISH was performed as previously published [54] using 1 μg/mL mouse monoclonal anti-GFP (isotype IgG2a, clone N86/8; NeuroMab, Davis CA).

### qRT-PCR

RNA was isolated from micro-dissected ROI of 10-week-old male WT mice or ground human brain tissue using TRIReagent Kit (ThermoFisher, Waltham MA). cDNA was synthesized with random hexamers from 1μg RNA using SuperScriptIV Reverse Transcriptase (ThermoFisher). Quantification of ErbB4 isoforms was performed as described [55] using TaqMan assays (ThermoFisher).

### Imaging and Quantification

FastRED fluorescent signal was excited at 530 nm and analyzed at 20x magnification. Unbiased automated signal detection and quantification was performed using CellProfiler [56]. Intensity threshold was determined based on background intensity in ErbB4-Δ2 KO sections and dot diameter threshold (≥ 3 pixels) based on mean dot diameter in WT sections. Dots/area, percentage of positive cells and average number of dots/cell were calculated.

### Statistical Analysis

All data represent the mean ± SEM and statistical significance was set at *p* < 0.05. Statistical analyses were performed using one-way ANOVA and Tukey’s multiple comparison test. Statistical analyses are tabulated in Supplemental Tables.

## Results

### Sensitivity and Specificity of the Novel Single-Pair Probe ISH Approach

**Fig. 2:**
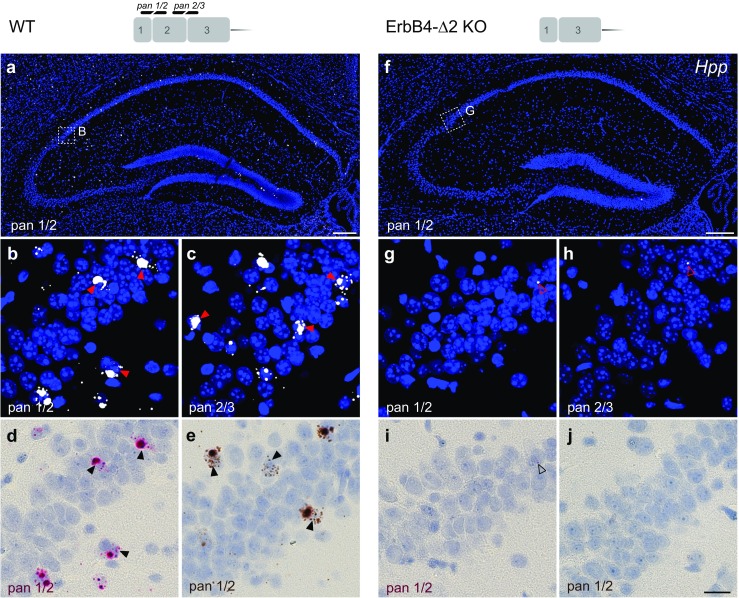
Single-pair probes targeting unique exon junctions are specific and sensitive. The specificity and sensitivity of single-pair probes targeting exon-exon boundaries were determined by hybridizing sections from WT (**a-e**) and ErbB4-Δ2 KO mice (**f-j**). Probes targeting the exon 1/2 (pan1/2; **a,b,d,e**) or exon 2/3 (pan 2/3; **c**) junctions—common to all ErbB4 isoforms—labeled scattered cells in the WT hippocampus (*arrowheads*). (**f-j**) By contrast, neither probe generated signals in sections from ErbB4-Δ2 KO mice (background signal marked by *open arrowheads*). (**b, g**) Magnified insets in panels **(a)** and **(f)** are from area CA2. Signal can be detected by alkaline phosphatase and FastRED visible both in fluorescence (**a-c, f-j**) and bright field microscopy (**d, i**) or horseradish peroxidase and diaminobenzidine (**e, j**). Scale bars: **a**, **f** 200 μm; **j** 20 μm

Initially, to determine if single-pair BaseScope probes targeting exon junctions provide the necessary sensitivity to detect ErbB4 transcripts, we hybridized sections of WT mice with two independent “panErbB4” single-pair probes that target mRNA junctions between exons 1/2 (pan 1/2) and exons 2/3 (pan 2/3) that are present in all receptor isoforms (see Fig. 1). The amplified signal was detected following alkaline phosphatase and FastRED staining using fluorescence (Fig. 2a–c) and bright-field microscopy (Fig. 2d), or following horseradish peroxidase and diaminobenzidine treatment (Fig. 2e; Fig. S1). In hippocampal sections from WT mice, both panErbB4 single-pair probes labeled scattered cells (Fig. 2a–e). This pattern is consistent with the expression pattern of ErbB4 obtained by 20 probe pairs in multiplex fluorescent ISH (Fig. S1N), the restricted expression of ErbB4 in GABAergic interneurons (Fig. S1O) and its absence in pyramidal neurons [57].

To validate the specificity of the single-pair probes, we used as negative controls sections from ErbB4-Δ2 KO mice that lack exon 2 [49], and targeted the upstream and downstream junctions of exon 2 with probes pan 1/2 and pan 2/3, respectively. In contrast to the high cellular ErbB4 expression in hippocampal interneurons of WT mice (Fig. 2a–e), the signal was absent in the ErbB4-Δ2 KO (Fig. 2f–j; Fig. S1). In summary, these results show the sensitivity and specificity of single-pair probes to visualize exon junctions.

### Semi-Quantitative Analysis of Junction-Specific Single-Pair Probe ISH

**Fig. 3:**
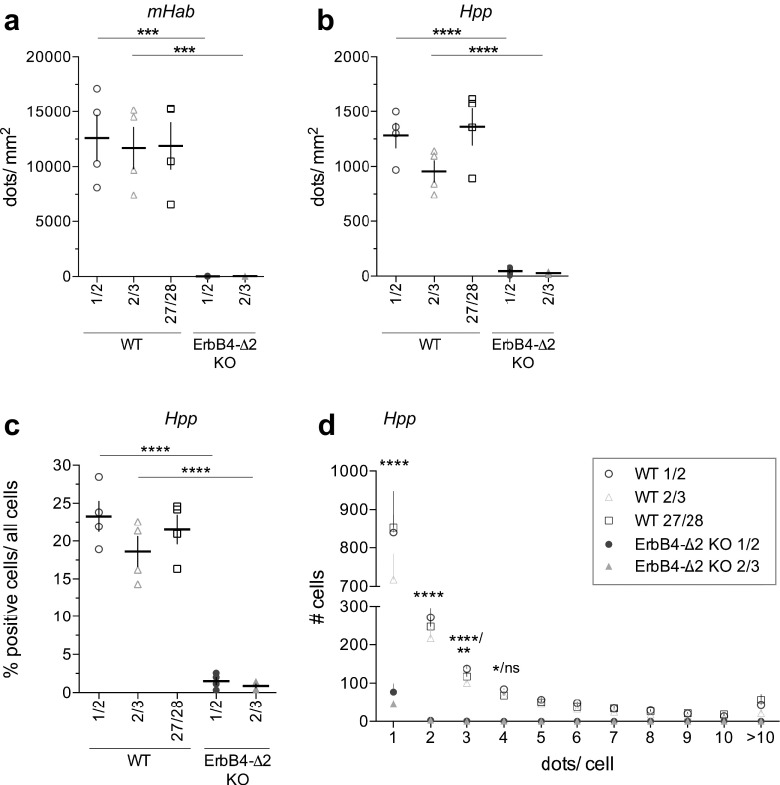
Detection levels for independent probes targeting distinct exon junctions are similar and differ markedly from background in ErbB4-Δ2 KOs. In situ hybridization signals of single-pair probes pan1/2 and pan 2/3 are significantly lower in sections from ErbB4-Δ2 KO mice compared to WT mice in the (**a**) medial habenula (*mHab*) and (**b**) hippocampus (*Hpp*) (*n* = 4; one-way ANOVA, see Table S1) and did not differ among pan 1/2, pan 2/3 and pan 27/28 probes in sections from WT mice. (**c**) Percentage of positive cells relative to all cells in WT hippocampus (CA1–CA3). (**d**) Histogram distribution of dots/ positive cell detected with single-pair panErbB4 probes in hippocampal CA1–CA3 on sections from WT and ErbB4-Δ2 KO mice. Significance shown for comparisons between WT 1/2 vs. KO 1/2 and WT 2/3 vs. KO 2/3, respectively (*n* = 4; two-way ANOVA, see Table S4). Adjusted *p* values according to Tukey’s multiple comparison test: *p < 0.05, **p < 0.01, ***p < 0.001, ****p < 0.0001

**Fig. 4:**
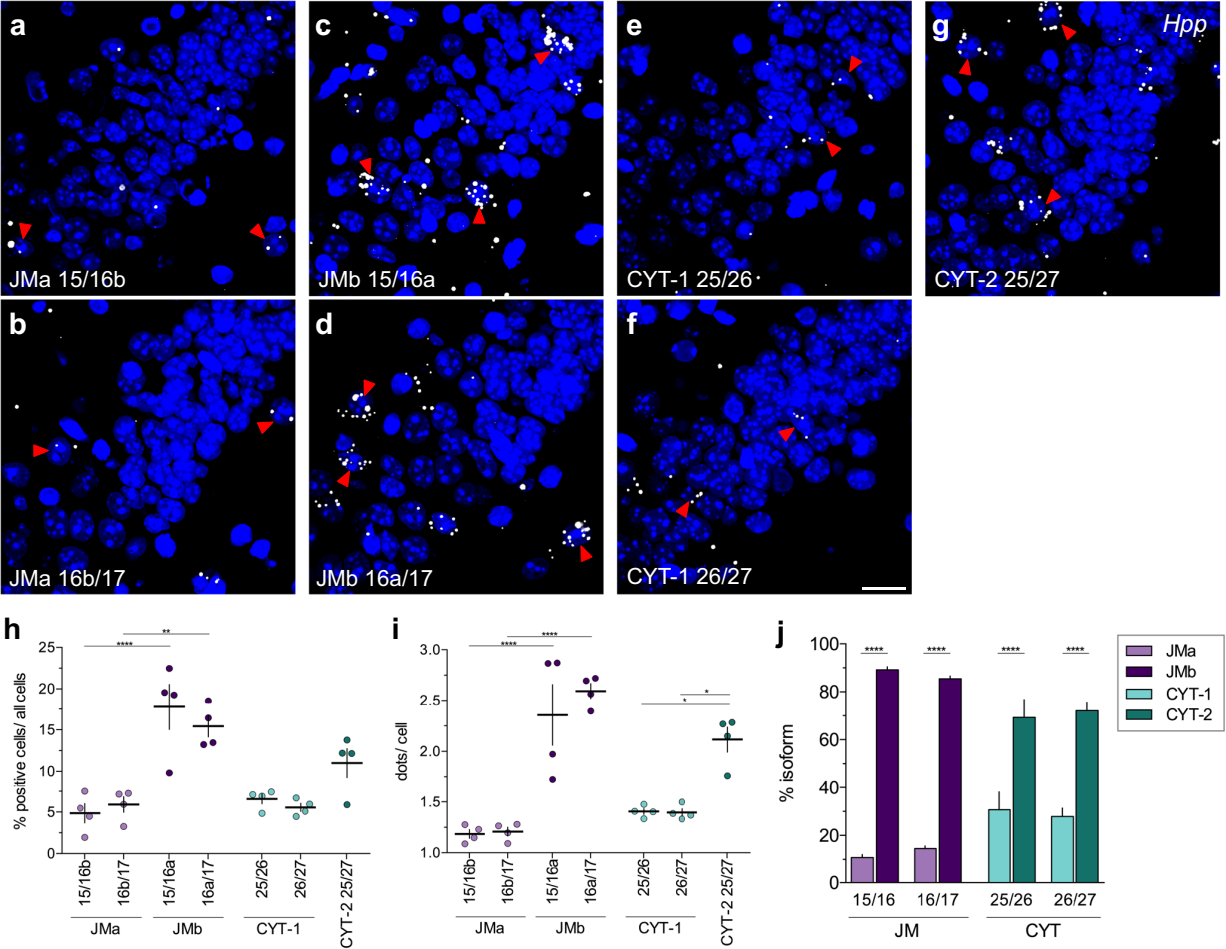
JMb- and CYT-2-containing transcripts are the major ErbB4 isoforms expressed in adult hippocampus. (**a**–**g**) Hybridization of ErbB4 isoform-specific single-pair probes in hippocampal CA2 area of WT mice. Arrowheads indicate examples of positive cells. (**h**, **i**) Percentages of positive cells/total cells and average dots/cell in hippocampal CA1–CA3 areas were quantified for each isoform-specific probe using CellProfiler. Results derived with probes targeting the same isoform were not significantly different (*n* = 4; one-way ANOVA, see Table S2). (**j**) Relative abundance of JMa/JMb (*purple*) and CYT-1/CYT-2 (*cyan*) isoforms in the hippocampus (*n* = 4; one-way ANOVA, see Table S2). Adjusted *p* values according to Tukey’s multiple comparison test: **p < 0.01, ****p < 0.0001. Scale bar: 20 μm

To complement our qualitative analysis, we wrote a pipeline (macro) for the open-source software CellProfiler [56] that allows for the unbiased quantification of signals. The pipeline, which is available online, identifies fluorescent FastRED signals above threshold and assigns them to the closest DAPI-positive nuclei. The results are exported in Excel-format (for details see Supplemental Information). Using this approach on sections from WT mice, we found that ErbB4 expression is uniformly high in the medial habenula (mHab; Fig. 3a) and that its overall regional levels are low in the hippocampus (Hpp; Fig. 3b), consistent with prior studies [58, 59]. Expression analysis at a cellular level in the hippocampus indicate that approximately 20% of cell nuclei are labeled by single-pair panErbB4 probes (Fig. 3c), as was expected from the known restricted expression of ErbB4 in cortical and hippocampal GABAergic interneurons (Fig. S1; [57]). Despite the low regional expression in the hippocampus, signals on sections from WT mice were dramatically higher than in sections from ErbB4-Δ2 KO mice using probes that target either boundary of the deleted exon 2 (Fig. 3b, c; *p* < 0.0001). Background levels in ErbB4-Δ2 KO (see open arrowheads in Fig. 2g–i) consisted mainly of single dots (Fig. 3d), whereas all probes targeting distinct ErbB4 exon boundaries on sections from WT mice were expressed notably above these background levels. Although hybridization efficiencies of small single-pair probes could theoretically vary depending on the targeted RNA sequence or be hindered by binding proteins or secondary structure, signals from single-pair probes targeting the 5′ end (pan 1/2, pan 2/3) and the 3′ end (pan 27/28; Fig. S1M) of the ErbB4 mRNA coding sequences were not different (Fig. 3a–d; Table S1, S4). Moreover, signals from single-pair probes targeting either 5′ or 3′ boundaries of each alternatively spliced exon did not differ (Fig. 4; Table S2), indicating sensitivities of single-pair probes are generally comparable; therefore, all subsequent analyses were performed with probes targeting the 5′ upstream exon boundaries of alternatively spliced exons.

### Differential Expression of ErbB4 Isoforms in Distinct Regions of the Adult Brain

**Fig. 5:**
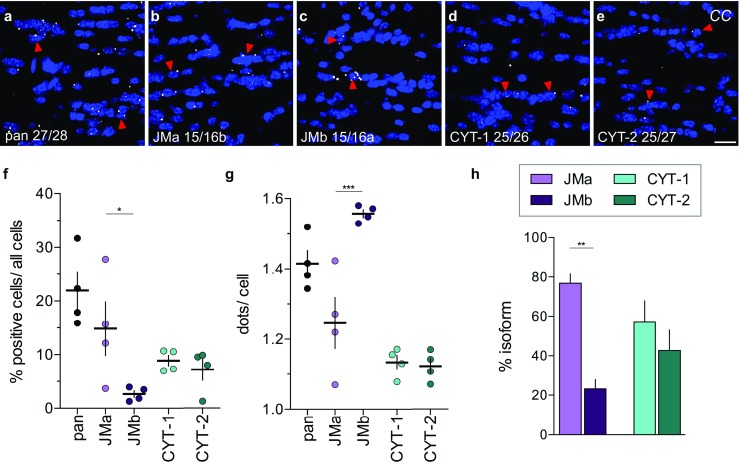
Pattern of ErbB4 JMa and CYT-1 isoform expression in the corpus callosum differ markedly from other brain areas. (**a**–**e**) Representative in situ hybridization images hybridized with pan and isoform-specific probes in the corpus callosum (*CC*). Arrowheads indicate representative positive cells. The (**f**) percentage of positive cells, (**g**) average number of dots/positive cell and (**h**) relative expression levels of ErbB4 JMa/JMb and CYT-1/CYT-2 isoforms were quantified using CellProfiler (*n* = 4; one-way ANOVA, *p < 0.05, **p < 0.01, ***p < 0.001, see Table S3). Scale bar: 20 μm

Next, we used single-pair probes targeting JMa/JMb and CYT-1/CYT-2 exons to analyze ErbB4 isoform distribution in the adult mouse hippocampus. We found that the non-cleavable juxtamembrane isoform JMb (> 85%) and the cytoplasmic isoform CYT-2 (~ 70%) are the predominant isoforms (Fig. 4j), consistent with qRT-PCR data (Fig. S2A). As in the hippocampus, JMb and CYT-2 also are the predominant ErbB4 isoforms in most brain areas, including the retrosplenial cortex and the reticular thalamic nucleus (Fig. S3). In stark contrast, in the corpus callosum, where total ErbB4 expression is relatively low compared to the aforementioned regions [58, 59], JMa (~ 75%) and CYT-1 (~ 55%) represent most of the receptor isoforms (Fig. 5). This novel observation is consistent with qRT-PCR using microdissected corpus callosal-enriched tissue (Fig. S2B); a similar expression pattern is found in the thalamus (Fig. S4). Interestingly, although in the corpus callosum the percentage of cells expressing JMa is higher than those expressing JMb (Fig. 5f; *p* = 0.0382), we observed higher JMb/cell than JMa/cell (Fig. 5g; *p* = 0.0006). Based on the varying expression patterns of JMa/JMb in the corpus callosum, we hypothesized that different cell-types in the corpus callosum express distinct ErbB4 JM isoforms.

### Expression of the Cleavable JMa Isoform in Cells of the Oligodendrocyte Lineage

**Fig. 6:**
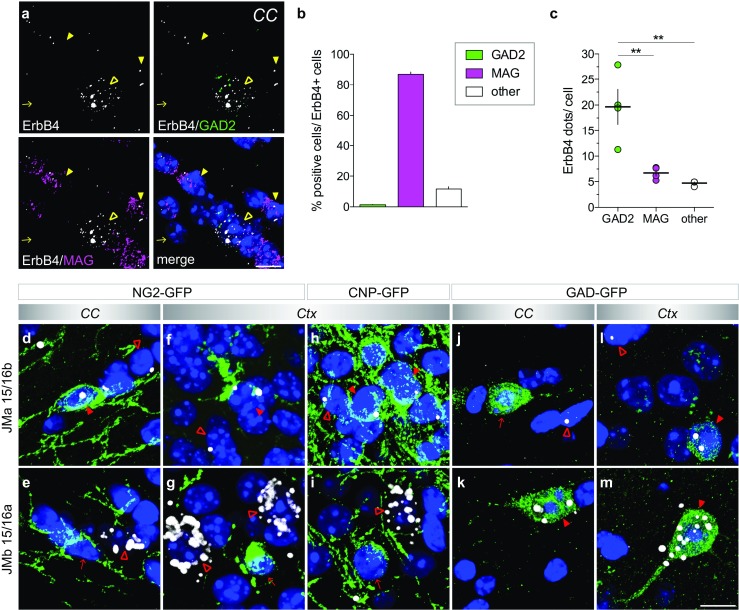
Oligodendrocytes and GABAergic neurons in the corpus callosum express different ErbB4 juxtamembrane isoforms. (**a**) Multiplex fluorescent in situ hybridization shows that ErbB4 (*white*) is expressed in both GAD2-positive GABAergic neurons (*green*; *open yellow arrowheads*) and MAG-positive oligodendrocytes (*magenta; yellow arrowheads*) in the corpus callosum (*arrow* ErbB4-negative cell). Note that dots are smaller compared to single-pair probe ISH, as signals are not enzymatically amplified. (**b**, **c**) Quantification of data shown in A (*n* = 4). (**b**) The majority of ErbB4+ cells in the corpus callosum co-expresses the oligodendrocytes marker MAG (86.95 ± 1.54%), whereas a small fraction is positive for the GABAergic marker GAD2 (1.40 ± 0.23%); 11.65 ± 1.48% of ErbB4+ cells were not labeled with either marker. (**c**) However, GABAergic neurons express higher levels of ErbB4 per cell than oligodendrocytes (19.65 ± 3.39 dots/cell vs. 6.73 ± 0.61 dots/cell, *p* = 0.0034; GAD2 vs. other 4.72 ± 0.23 dots/cell, *p* = 0.0013 *n* = 4; MAG vs. other *p* = 0.7614; F(2,9) = 16.53, *p* = 0.001; one-way ANOVA; Tukey’s multiple comparisons test: **p < 0.01). (**d**–**m**) Isoform-specific in situ hybridization using probes JMa 15/16b (**d**, **f**, **h**, **j**, **l**) and JMb 15/16a (**e**, **g**, **i**, **k**, **m**) was combined with post hoc immunohistochemistry for GFP (*green*) on sections from NG2-GFP (**d**–**g**), CNP-GFP (**h**, **i**) and GAD-GFP (**j**–**m**) transgenic mice. JM isoforms (*white*) were detected on GFP+ cells (*red arrowheads*), as well as on GFP negative cells (*open red arrowheads*) in the corpus callosum (*CC*) and the cortex (*Ctx*). Arrows depict GFP+ cells negative for JM probes. Note that the detection of JM isoforms in the corpus callosum of CNP-GFP mice was not possible because of the high density of GFP+ myelin sheaths [50]. Scale bar: 10 μm

To investigate the aforementioned hypothesis, we began by using multiplex fluorescent ISH (RNAscope) to analyze the cell-type specific expression of ErbB4 in the corpus callosum and found that both GAD2-positive GABAergic neurons and MAG-positive oligodendrocytes express the receptor (Fig. 6a). Interestingly, oligodendrocytes comprised the majority (~ 85%) of ErbB4-expressing cells, but express lower amounts of ErbB4 than GABAergic neurons (Fig. 6b, c; *p* = 0.0034).

A present limitation of the novel single-pair probe ISH approach described here, in contrast to the multiplex system, is that its amplification chemistry is limited to one fluorescent/colorimetric channel per section and, does not allow for the simultaneous detection of independent probes with distinct fluorophores (e.g. ErbB4 exon-specific single-pair probe and cell marker probe such as MAG). To circumvent this limitation, first we had to develop a post hoc immunohistochemical protocol because most of antibody cell markers tested were not compatible with the fixation and latter permeabilization protocol (i.e., protease treatment) necessary for ISH—even on fresh frozen sections that allow for milder pretreatment conditions than formalin-fixed paraffin sections. However, we identified a GFP antibody that is compatible with this ISH procedure and has the advantage that it is of broad use for other studies. Next, to unambiguously determine the cell-type expressing JMa transcripts, we used transgenic mice expressing GFP under specific promoters for GABAergic neurons (GAD) or for precursor (NG2) and mature (CNP) oligodendrocytes (details see Materials and Methods). Interestingly, we found that ErbB4 JMa isoforms are expressed in NG2+ oligodendrocyte precursor cells (OPCs) in the corpus callosum and cortex (Fig. 6d, f), as well as in CNP-GFP+ oligodendrocytes in the cortex (Fig. 6h); JMb isoforms were not detected in neither of these cell-types (Fig. 6e, g, i). Consistent with our hypothesis, GABAergic neurons in the corpus callosum and neocortex expressed high levels of JMb (Fig. 6k, m), but low amounts of JMa isoforms (Fig. 6j, l). Taken together, these findings confirm that the cleavable juxtamembrane isoform JMa is the major, if not the sole, juxtamembrane isoform expressed in cells of the oligodendrocyte lineage, whereas JMb transcripts are predominant in GABAergic neurons.

### Conservation of Differential ErbB4 Isoform Expression in Human Cortex and Corpus Callosum

**Fig. 7:**
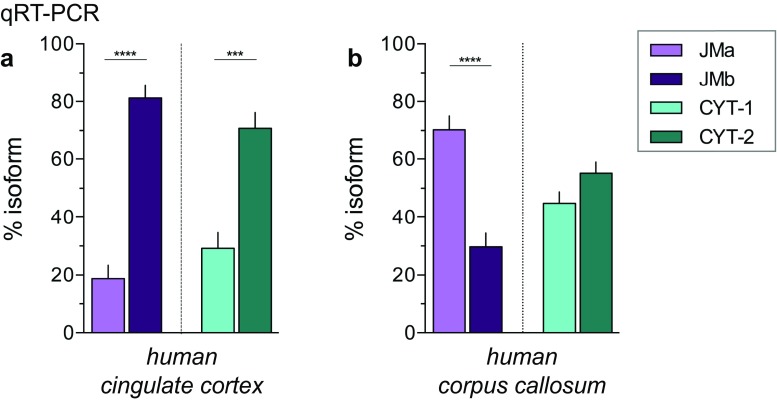
Distinct patterns of ErbB4 JM and CYT isoforms in the gray and white matter are conserved between humans and mice. Relative abundance of JMa/JMb (*purple*) and CYT-1/CYT-2 (*cyan*) isoforms in the adult human cingulate cortex (**a**) and corpus callosum (**b**) was determined by TaqMan qRT-PCR (n = 4; one-way ANOVA, see Table S5). Adjusted *p* values according to Tukey’s multiple comparison test: ***p < 0.001, ****p < 0.0001

Finally, to evaluate the relevance of the cell-type-specific expression of ErbB4 JM isoforms in humans, we analyzed the relative abundance of ErbB4 isoforms in the cingulate cortex and corpus callosum by qRT-PCR from human RNA samples. As in the adult mouse, ErbB4 JMb and CYT-2 were the major ErbB4 isoforms in the human cingulate cortex (~ 80 and ~ 70%, respectively; Fig. 7a). Importantly, in the corpus callosum JMa was predominant (~ 70%) and equal amounts of CYT were detected (Fig. 7b). This suggests that the cell-type-specific ErbB4 isoform expression is conserved from mouse to human, and that cleavable JMa ErbB4 is the predominant ErbB4 isoform in human oligodendrocytes.

## Discussion

Here, we demonstrate the use of a novel sensitive non-radioisotropic ISH approach, called BaseScope, to analyze exon junctions in tissue sections at a single-cell level that has universal applicability to study short RNA sequences—including splice variants in the brain and other tissues. We carefully validate the sensitivity and specificity of junction-specific probes used for this ISH approach, and show that single-pair probes are generally comparable. Moreover, the semi-quantitative results obtained are consistent with established isoform analyses using TaqMan qRT-PCR. By using this novel ISH approach that provides cellular resolution, we identified differential regional ErbB4 isoform expression in the adult mouse brain that is conserved in humans, and that results from the predominant cell-type-specific expression of juxtamembrane isoforms in neurons (JMb) and cells of the oligodendrocyte lineage (JMa).

### Differential and Cell-Type-Specific Expression of ErbB4 Isoforms in the Adult CNS

Our analyses identified ErbB4 transcripts harboring the JMb and CYT-2 exons as the two major isoforms in most adult mouse brain areas (e.g. hippocampus, cortex, reticular thalamic nucleus); in line with other studies analyzing ErbB4 isoform expression in the different brain areas across species—including humans [37–39, 60–63]; but see [41]. Taking advantage of the expression overview of ErbB4 isoforms by single-pair probe ISH, we identified brain regions where—although generally low—ErbB4 JMa and CYT-1 isoforms comprise most ErbB4 expressed, namely the corpus callosum and thalamus. Of note, the exclusive detection of JMa ErbB4 isoforms in the oligodendrocyte lineage (Fig. 6) is entirely consistent with a recent study that found this distribution of ErbB4 by using RNAseq from cell-sorted brain cells [4]. The fact that JMa, but not JMb, isoforms are cleaved by metalloproteases, which is a requirement for intramembranous gamma-secretase cleavage that releases a transcriptionally active ICD [38, 43, 44], raises the possibility that NRG/ErbB4 signaling uniquely regulates oligodendrocyte maturation through ErbB4-dependent transcriptional mechanisms. Consistent with the expression of ErbB4 in oligodendrocytes, previous studies have reported a role of NRG/ErbB signaling in glial development and myelination [64–67].

### Alterations of ErbB4 Isoform Expression in Scz

Whereas JMa and CYT-1 are the minor ErbB4 isoforms in the adult brain (this study; [37, 38]), they have been repeatedly reported to play an important role during neurodevelopment [68–70] and higher expression of JMa and CYT-1 ErbB4 isoforms has been reported in postmortem DLPFC of Scz patients independently by several groups [29, 39–41]. This is interesting considering the increased expression of disease-associated genes in neurodevelopmental disorders during fetal development [71, 72] and high NRG1 expression at ages with highest risk for Scz onset [73, 74]. Further it raises the question whether the increased expression of JMa and CYT-1 isoforms in the DLPFC of Scz results from alterations in the expression or number of cells from the oligodendrocyte lineage and/or a switch in ErbB4 isoform expression in GABAergic neurons. A proposed role of oligodendrocytes and myelination deficits associated with Scz has been emerging (see [75]). An ErbB4 SNP was shown to affect brain white matter integrity [76], subcortical white matter is lost in Scz patients [77, 78], and genes related to oligodendrocyte function have been associated with Scz [79, 80]. These observations are interesting in the context of our novel finding that OPCs and oligodendrocytes express predominantly or exclusively the ErbB4 JMa isoform. On the other hand numerous postmortem studies implicate alterations in GABAergic neurons in the DLPFC and hippocampus of persons with Scz [81, 82], where a reduction of GABAergic neuron markers [83] in particular those associated with fast-spiking interneurons [84, 85], has been frequently reported. Interestingly, the changes have been proposed to occur in specific subtypes of interneurons [39, 40]. Future studies, using ErbB4 isoform-specific single-pair probes reported here, will be important to investigate ErbB4 JMa/JMb and CYT-1/CYT-2 ratios in postmortem human brains of Scz patients and controls to precisely identify the cell-type(s) that underlie the changes in ErbB4 isoforms. Because in addition to ErbB4 the alternative splice variants of many at-risk genes are frequently aberrant in Scz [12] and affective, addictive and autism spectrum disorders [11], single-pair probe ISH at a cellular level could generally advance our understanding of isoform changes in psychiatric disorders.

### General Considerations for the Broad Application of the Single-Pair Probe ISH Approach

This study is the first to analyze exon junctions using a fluorescent ISH assay. This approach is not limited to splice variants studies, but could be generally used to analyze short mRNA sequences (e.g. pre-miRNAs and snoRNAs), highly homologous transcripts and circular RNAs, as well as point mutations. In addition, the freely-available automated analytic tool developed here renders this ISH approach a valuable semi-quantitative tool to analyze expression at a single-cell level, which complements other quantitative methodologies such as qRT-PCR and RNAseq analysis to study splice variants. However, single-probe ISH (BaseScope) has the added benefit of post-assay analyses in morphological conserved tissue. Using post-hoc immunohistochemical analysis following hybridization of single-pair probes on sections of transgenic mice, we show how to overcome the current single-plex platform limitation to identify the cell-types expressing specific splice variants. Of note, the anti-GFP antibody used herewith is one of few antibodies (< 10%) compatible with protease permeabilization.

Altogether, the advances of this novel ISH approach in analyzing short sequences and isoforms at cellular resolution in the tissue environment by far outweigh a few limitations or difficulties of this technology that merit to be mentioned. Probes targeting highly abundant transcripts tend to produce signal accumulations (clumps) during the enzymatic conversion of FastRED (see Fig. 2b–e; Fig. S1). As shown earlier (Figs. 3 and 4), in our experience hybridization efficiencies between unrelated single-pair probes are in general extremely similar but on occasion, as was the case of CYT probes, can give weaker signals relative to the panErbB4 or juxtamembrane single-pair probes (compare Figs. 3a and 4h); the differences observed could have resulted from intrinsic differences of the targeted mRNA sequences (i.e., looping). Therefore, quantification using this novel single-pair ISH should be considered carefully. Nevertheless, the relative signals for CYT-1/CYT-2 isoforms were conserved as confirmed by qRT-PCR analysis (Fig. 4j, S3A), supporting the semi-quantitative nature of this approach.

Taken together, our study underscores the important and unique utility of this novel single-pair probe ISH technique to investigate, with cellular resolution in tissues, the expression of short and highly homologous RNA sequences. As discussed above, whilst BaseScope should be considered as semi-quantitative approach, it can be used to complement other traditionally used methodologies like qRT-PCR and RNAseq. Its numerous applications renders the single-pair probe ISH as an indispensable tool to advance studies on mRNA regulation and complexity, and their association with numerous neurological and psychiatric diseases.

## Electronic Supplementary Material

ESM 1 (DOCX 29030 kb)
