# Supplementary material for: A Novel Ultrasensitive In Situ Hybridization Approach to Detect Short Sequences and Splice Variants with Cellular Resolution
Source: Mol Neurobiol. 2017 Dec 20;55(7):6169–81. doi: 10.1007/s12035-017-0834-6 (PMC5994223; doi:10.1007/s12035-017-0834-6)
Supplement: Supplementary file 1 — (DOCX 29030 kb) [file 12035_2017_834_MOESM1_ESM.docx]

**A novel ultrasensitive *in situ* hybridization approach**

**to detect short sequences and splice variants**

**with cellular resolution**

***Supplemental Information***

**Supplemental Materials and Methods**

**Supplemental Figures S1-S4**

**Supplemental Table S1-S5**

**Supplemental Materials And Methods**

**Animals.** Homozygous ErbB4 knock-out (KO) mice lacking exon 2 were rescued from embryonic lethality by transgenic ErbB4 overexpression in the heart[1], and will be hereafter designated as ErbB4-Δ2 KO mice. CNP-mEGFP, hereafter referred to as CNP-GFP[2] (<https://www.jax.org/strain/026105>), NG2-mEGFP/ Cspg4-mEGFP, hereafter referred to as NG2-GFP[3] (<https://www.jax.org/strain/022735>), and wild-type (WT) C57BL/6J mice (<https://www.jax.org/strain/000664>) were obtained from the Jackson Laboratory (Bar Harbor, ME). GAD67-GFP mice, hereafter referred as GAD-GFP mice[4], were a kind gift from Yuchio Yanagawa (Gunma University, Japan). Mice were kept on a 12-12h light-dark schedule with access to food and water *ad libitum* and handled in accordance with the National Institutes of Health (NIH) Animal Welfare guidelines. All animal procedures were approved by the NIH Animal Care and Use Committee. Ground human brain samples from four male adult control individuals (age 44-53) were obtained from the Human Brain Collection Core at NIMH.

**Tissue preparation for *in situ* hybridization.** Ten-week-old adult mice of both sexes were transcardially perfused with 4% paraformaldehyde (Electron Microscopy Sciences, Hartfield PA) in 0.1 M PBS, pH 7.4. Dissected brains were post-fixed overnight in 10% neutral buffered formalin (Sigma-Aldrich, St. Louis MO) at 4°C. Tissue was embedded in paraffin after ethanol dehydration steps followed by xylene. Serial coronal paraffin sections (8 μm) were mounted on Superfrost slides (Daigger, Vernon Hills IL) and baked for 10 min at 70°C.

***In situ* hybridization (ISH).** The novel junction-specific ISH approach, known as BaseScope, is based on the same principles than the well-established multiplex fluorescent ISH RNAscope^®^ (Advanced Cell Diagnostics, Newark, CA [5]). The high specificity of both ISH technologies is achieved from the unique design of probes, called ‘ZZ’ probe pairs, consisting of two 18-25bp antisense probes, a spacer region and a 14bp tail that is necessary for signal amplification. The tail region is recognized by a preamplifier that can only bind if both ‘Z’ probes in a pair are hybridized directly adjacent to each other, suppressing off-target non-specific hybridization and thus resulting in extremely low background [5]. The sensitivity of BaseScope is increased by several amplification steps generating an amplification ‘tree’. The additional enzymatic and amplification steps in BaseScope ISH allow the use of a single ‘ZZ’ probe pair, instead of the 6-20 ‘ZZ’ probe pairs necessary in RNAscope, for signal detection. It is these properties that make BaseScope suitable to detect short nucleotide sequences, such as exon junctions, to analyze expression of alternative spliced transcripts. BaseScope probes are comprised of 18-25bp oligonucleotide sequences designed by a proprietary algorithm to meet required melting temperature for assay hybridization conditions and to avoid cross-hybridization. One oligonucleotide probe hybridizes target sequences across the exon junction and the other probe to immediately adjacent region. Targeted sequences of customized junction-specific ErbB4 ISH probes are listed in Table 1 and schematically illustrated in Fig. 1. RNAscope^®^ probes were ErbB4 (Mm-ErbB4; Cat No. 318721), GAD-2 (Mm-GAD2-C2; Cat No. 415071-C2) and MAG (Mm-MAG-C3; Cat No. 446451-C3). Both RNAscope^®^ and BaseScope ISH assays were performed on 8 μm-thick formalin-fixed paraffin embedded (FFPE) sections. Briefly, FFPE sections were incubated for 1h at 60°C, subsequently deparaffinized by two washes in xylene for 5 min at room temperature (RT), and washed twice for 3 min in ethanol. To quench endogenous peroxidase activity, dried sections were incubated with H_2_O_2_ treatment for 10 min at RT. Target retrieval at 100°C for 15 min was found to be optimal for adult mouse brain sections, followed by treatment with Protease III for 30 min at 40°C. After pretreatment, sections were thoroughly washed and incubated with probes for 2 h at 40°C. For multiplex fluorescent ISH, sections were incubated with amplification solutions (AMP) as follows: AMP1, 30 min at 40°C; AMP2, 15 min at 40°C; AMP3, 30 min at 40°C; and AMP4B, 15 min at 40°C. For FastRED detection of junction-specific ISH, sections were incubated as follows: AMP0, 30 min at 40°C; AMP1, 15 min at 40°C; AMP2, 30 min at 40°C; AMP3, 30 min at 40°C; AMP4, 15 min at 40°C; AMP5, 30 min at RT; AMP6, 15 min at RT; and FastRED (60:1 mixture of FastRED A and B solution), 10 min at RT. Sections were washed thoroughly twice for 2 min in-between steps with washing buffer. Sections were counterstained with DAPI (1 μg/mL in PBS; Thermo Fisher, Waltham MA) for 30 sec and mounted with Mowiol-DABCO. Sections shown in Fig. 2D,I and Fig. S1C,D,I,J were additionally counterstained with haematoxylin (Electron Microscopy Sciences).

**Immunostainings.** Post-hoc GFP immunohistochemistry (IHC) was performed immediately following ISH as previously published[6]. Briefly, sections were washed three times in 0.1 M PBS for 5 min each and blocked with 10% normal donkey or goat serum (Sigma-Aldrich) in 0.1 M PBS with 0.3% Triton X-100 (ThermoFisher) for 1h at RT. Sections were incubated with 1μg/mL mouse monoclonal anti-GFP (isotype IgG2a, clone N86/8; NeuroMab, Davis CA) in blocking solution overnight at 4°C. Following three washes with 0.1 M PBS + 0.25% Triton X-100, sections were incubated with donkey anti-mouse Alexa488 secondary antibody (Invitrogen A-21202, Thermo Fisher) in blocking solution for 2h at RT. Samples were extensively washed with 0.1 M PBS, counterstained with DAPI and mounted with Mowiol-DABCO.

**Quantitative Real-Time PCR.** RNA was isolated from micro-dissected tissue from hippocampus, thalamus and corpus callosum of five ten-week-old male WT mice, and from micro-dissected ground tissue of human cingulate cortex and corpus callosum from four individuals using the TRI Reagent Kit (Thermo Fisher). cDNA synthesis was synthesized in a total volume of 20μl according to manufacturer’s protocol, using 1 μg RNA template, SuperScript IV Reverse Transcriptase (Thermo Fisher) and random hexamers for 20 min at 55°C. qRT-PCR of ErbB4 isoforms was performed using custom-made TaqMan assays (Thermo Fisher). Flanking primers and TaqMan probes were as follows (all sequences correspond to the sense strand):

|  | **Mouse** | | **Human** | |
| --- | --- | --- | --- | --- |
| **Assay** | **Primers** | **TaqMan probe** | **Primers** | **TaqMan probe** |
| **JMa** | 5’CCACCCTTGCCATCCAAA3’  5’CCAATGACTCCGGCTGCAATCA3’ | FAM-ATGGACGGGCCATTCCACTTTACCA-MGB | 5’CCACCCATGCCATCCAAA3’  5’CCAATTACTCCAGCTGCAATCA3’ | FAM- ATGGACGGGCCATTCCACTTTACCA -MGB |
| **JMb** |  | FAM- TTCAAGCATTGAAGACTGCATCGGCCTGAC-MGB |  | FAM- CTCAAGTATTGAAGACTGCATCGGCCTGAT-MGB |
| **CYT-1** | 5’CAACATACCTCCTCCCATCTACAC3’  5’GCATTCCTTGTTGTGTAGCAAA3’ | FAM-TGAAATTGGACACAGCCCTCCTCCTG-MGB | 5’CAACATCCCACCTCCCATCTATAC3’  5’ACACTCCTTGTTCAGCAGCAAA 3’ | FAM- TGAAATTGGACACAGCCCTCCTCCTG-MGB |
| **CYT-2** |  | FAM-AATTGACTCCAATAGGAATCAGTTTGTGTACCAAGAT-MGB |  | FAM- AATTGACTCGAATAGGAACCAGTTTGTATACCGAGAT-MGB |
| **β-actin** | 5’ATCTGGCACCACACCTTCTACAAT3’  5’CCGTCTCCGGAGTCCATCA3’ | VIC-TGACCCAGATCATGTTTGAGACCTTCAACAC-MGB | 5’ATCTGGCACCACACCTTCTACAAT3’  5’CCGTCACCGGAGTCCATCA3’ | VIC- TGACCCAGATCATGTTTGAGACCTTCAACAC-MGB |

1ng cDNA was amplified using 0.25 μM isoform-specific FAM-labeled TaqMan probes and 0.9 μM corresponding primers (Thermo Fisher) in a total volume of 10 μl total volume using TaqMan universal PCR Master Mix (Thermo Fisher). As reference, β-actin was detected with a custom-made VIC®-labeled probe. Cycling was performed in 384-well plates using a QuantStudio 6 Thermocycler (Thermo Fisher) and the following parameters: 2min at 50°C and 10min at 92°C, followed by 40 cycles of 15s at 95°C, 1min at 60°C (for JM probes) or 65°C (for CYT probes). Standard curves (1fg–1ng) of cloned DNA for ErbB4 JMa/CYT-1 and JMb/CYT-2, as well for β-actin, were run beforehand to verify that sample values were in the linear range and that PCRs showed similar efficiency between isoform-specific assays. As negative controls, 100 pg DNA of non-matching isoforms were included to demonstrate assay specificity.

**Imaging and Quantification.** FastRED fluorescent punctuate signal (syn: “dots” and “puncta”) was analyzed on a Zeiss LSM710 confocal microscope at 20x and 63x magnifications using a 530 nm laser. Bright-field images were taken on a Zeiss Axiovert200 with an Axio Cam HRc at 63x magnification. For visualization, images were adjusted for overall brightness and contrast using Image J (http://imagej.nih.gov/ij/); fluorescent signal were converted into gray scale. For quantification, areas of interest were imaged in Z across the whole thickness of the section at 20x magnification and 1024x1024 resolution. Due to smaller puncta size, multiplex fluorescent ISH analysis (Fig. 6A) was performed on images acquired at 63x magnification. Image stacks were projected in Z using the maximum intensity method and then converted to RGB format or single channel images in case of the multiplex fluorescent ISH. ROIs were manually defined and measured using Image J; area size did not differ between groups analyzed (see Table S1-S3). Quantification was performed using CellProfiler[7], the pipelines (macros) are available at the provider’s homepage (cellprofiler.org/examples/published_pipelines). Intensity threshold was set based on the mean background intensity in all ErbB4-Δ2 KO sections and defined as 10x mean intensity. Then, dot diameter threshold was set as ≥3 pixels based on the mean dot diameter from all WT hippocampi analyzed (3.9 pixels); these settings were found to faithfully identify dots as manually verified in a subset of ROIs from both WT and ErbB4-Δ2 KO sections. Percentage of positive cells ((positive cells/ all cells) x 100), average number of dots/ area (in mm^2^) or dots/ cell were calculated. For multiplex fluorescent ISH, percentage of ErbB4-positive cells also positive for GAD2 or MAG ((marker/ ErbB4+ cells) x 100) and ErbB4 dots/ cell was calculated (Fig. 6B,C). Overlapping neighboring cells were excluded from the analysis. Hippocampal dentate gyrus was excluded from quantification of cellular analyses because the density of granule cells prevented accurate designation of DAPI-labeled nuclei.

**Statistical Analysis.** Population (n) in all analyses was defined as number of animals/humans analyzed. For histological analyses (Fig.2-5), four 10-week old mice were analyzed per group (WT: 4 males; ErbB4-Δ2 KO: 2 males and 2 females), a population size consistent with earlier studies[8]. ROIs were analyzed bilaterally on one brain section and cellular analyses comprised, depending on cell density, between 750 cells and 15,000 single cells per animal and ROI. All data represent the mean ± SEM and statistical significance was set at p<0.05. Statistical analyses were performed with Graph Pad Prism 6 using one-way ANOVA and Tukey’s multiple comparison test. Statistical significance (p values) are stated in the text; all values (including means ± SEM, degrees of freedom and multiple comparisons) are listed in Tables S1-S5. Two-way ANOVA analysis was used for the dots/ cell histogram distribution analysis shown in Fig. 3D (see Table S4).

**Figure S1. Visualization of exon-specific and multiplex ISH signal by fluorescent and chromogenic dyes in hippocampal GABAergic interneurons.** Hybridization of single-pair probes targeting exon 2 (*pan 1/2* and *pan 2/3*) in sections from WT **(A-F)** and ErbB4-Δ2 KO mice **(G-L)** was visualized using alkaline phosphatase and FastRED in fluorescence **(A,B,G,H)** or bright field microscopy **(C,D,I,J)** or horseradish peroxidase and diaminobenzidine **(E,F,K,L)**. **(M)** Hybridization with probe pan 27/28 targeting the 3’ end of ErbB4 transcripts showed essentially the same pattern (*arrowheads* – positive cells; *open arrowheads -* background signal). **(N,O)** Multiplex fluorescent ISH (20 probe pairs) shows that **(O)** ErbB4 (*white*) is expressed in scattered cells in the hippocampus that correspond to GAD2-positive (*green*) GABAergic neurons (*arrowheads*). Note that more transcripts are detected by the multiplex fluorescent ISH due to the increased sensitivity resulting from multiple probe pairs. Differences in dot sizes between the two assays are attributed to the differences in detection methodology (i.e. catalytic in the new junction-specific assay vs. fluorescent in the multiplex fluorescent ISH assay). Scale bars: **N** 200 μm; **L,M,O** 20 μm.

**FigureS2. TaqMan qRT-PCR analysis of ErbB4 isoforms in the adult mouse hippocampus (*Hpp*) and corpus callosum (*CC*).** Relative abundance of JMa/JMb (purple) and CYT-1/CYT-2 (cyan) isoforms was analyzed in the micro-dissected tissue of adult mouse hippocampus **(A)** and corpus callosum **(B)** by TaqMan qRT-PCR (n=5; one-way ANOVA, see Tables S2,S3). Adjusted p values according to Tukey’s multiple comparison test: **p<0.01, ****p<0.0001 (Tukey’s multiple comparison test).

**Figure S3. ErbB4 isoform expression pattern in the retrosplenial cortex and the thalamic reticular nucleus are similar to the hippocampus.** Representative images of pan and isoform-specific ErbB4 single-pair probe hybridizations in **(A-E)** the retrosplenial cortex (*Rsc*) and in **(F-J)** the thalamic reticular nucleus (*Rtn*); boxed areas are magnified in the insets shown on the bottom right of each panel. Representative positive cells are indicated (*arrowheads*). **(K,L)** Percentages of positive cells and relative expression of ErbB4 JMa/JMb and CYT-1/CYT-2 isoforms are quantified in the **(K)** Rsc (n=3) and **(L)** Rtn (n=4; one-way ANOVA; *p<0.05; see also Table S3). Scale bars: 50 μm (overviews); 10 μm (insets).

**Figure S4. As in the corpus callosum, JMa and CYT-1 isoforms are the major ErbB4 variants expressed in the thalamus. (A-E)** Representative *in situ* hybridization images hybridized with pan and isoform-specific single-pair probes in the medial thalamus (*Thal)*. Arrowheads indicate representative positive cells. The **(F)** percentage of positive cells, **(G)** average number of dots/ positive cell and **(H)** relative expression levels of ErbB4 JMa/JMb and CYT-1/CYT-2 isoforms were quantified using CellProfiler (n=4; one-way ANOVA, *p<0.05, ***p<0.001, see also Table S3). Scale bar: 20 μm.

**Table S1. Quantification and statistical analysis of ErbB4 expression in the medial habenula (mHab) and the hippocampus (Hpp) using pan ErbB4 single-pair probes.**


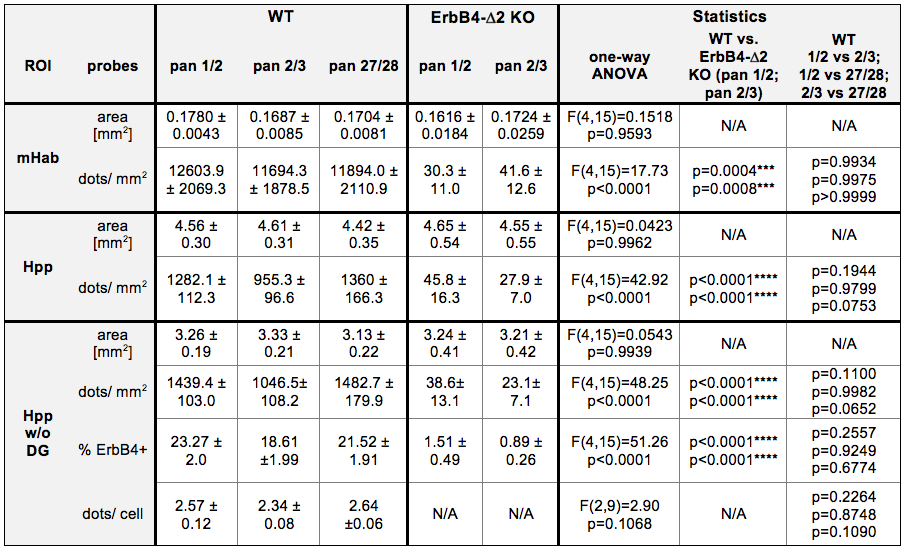


Values represent the mean ± SEM of analyzed areas, dots/ area and percentage of ErbB4-positive cells in sections of WT and ErbB4-Δ2 KO mice. *p<0.05, **p<0.01, ***p<0.001, ****p<0.0001 (n=4; one-way ANOVA with Tukey’s multiple comparison test). N/A: not applicable.

**Table S2. Quantification and statistical analysis of ErbB4 expression in the adult hippocampus (Hpp) using isoform-specific single-pair probes.**


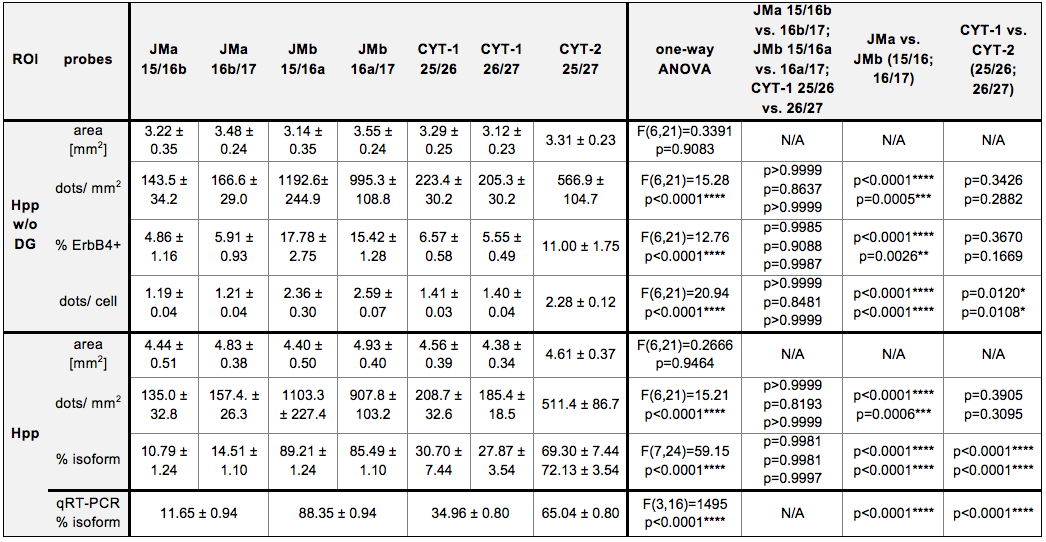


Relative isoform expression levels of JM and CYT isoforms (% isoform) in the Hpp (w/ or w/o the dentate gyrus (DG)), as determined by ISH and qPT-PCR. Values represent the mean ± SEM (n=4) of analyzed areas, dots/ area and percentage of ErbB4-positive cells, as well as dots/cell (analysis only performed for on hippocampus w/o DG). *p<0.05, **p<0.01, ***p<0.001, ****p<0.0001 (n=4; one-way ANOVA with Tukey’s multiple comparison test). N/A: not applicable.

**Table S3. Quantification and statistical analysis of ErbB4 isoform expression in various adult brain region using isoform-specific single-pair probes.**


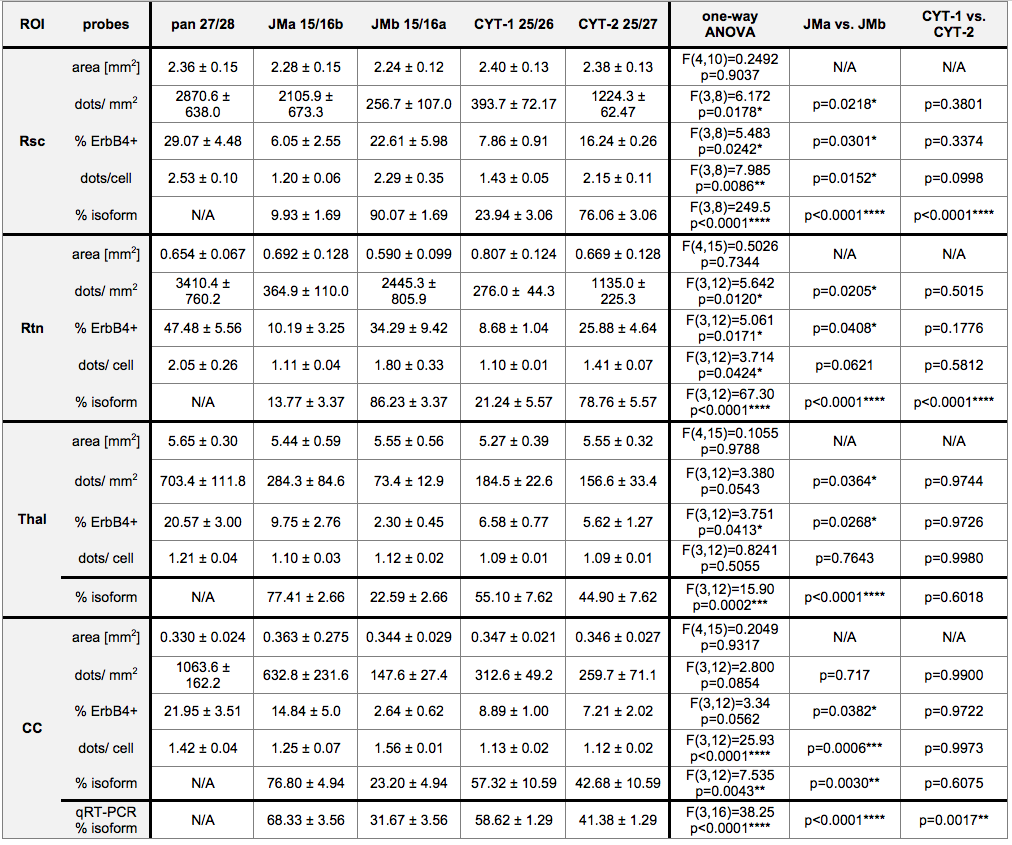


Values represent the mean ± SEM (n=3, 4) of analyzed areas, dots/ area, percentage of ErbB4-positive cells, dots/ positive cell and relative isoform expression (% isoform) in the retrosplenial cortex (Rsc), reticular thalamic nucleus (Rtn), thalamus (Thal) and corpus callosum (CC) hybridized with pan and isoform-specific single-pair ErbB4 probes. Relative isoform expression in the CC was additionally performed by qRT-PCR using TaqMan probes. One-way ANOVA was performed to compare isoform expression (except for area) was performed and adjusted p values of Tukey’s multiple comparison test are listed, *p<0.05, **p<0.01, ***p<0.001, ****p<0.0001. N/A: not applicable.

**Table S4. Histogram distribution of number of cells with same amount of dots in hippocampus of WT and ErbB4-Δ2 KO mice**.


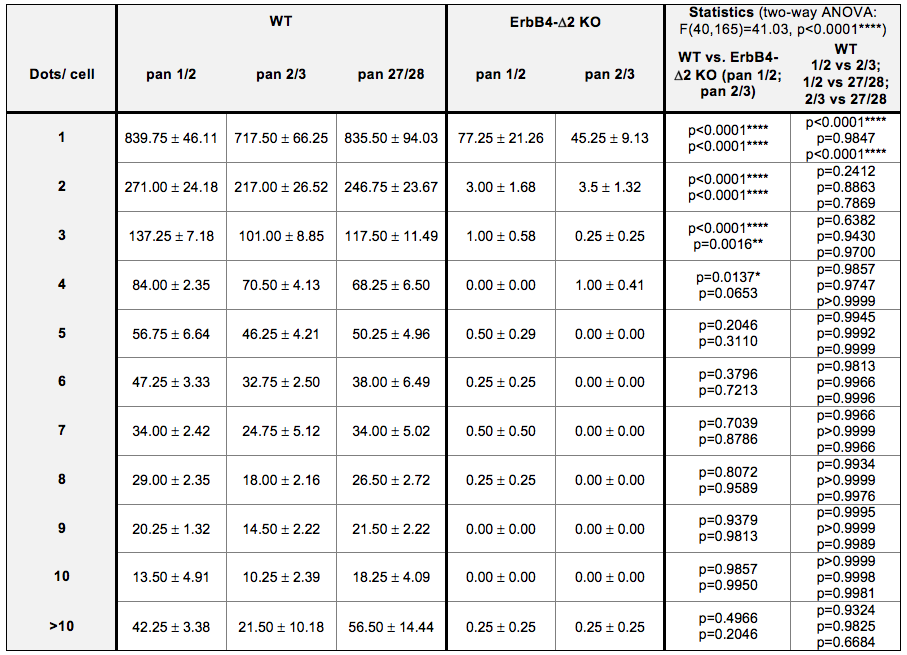


Mean values ± SEM are tabulated for the number of cells with between 1 and >10 dots/ cell. Adjusted p values are listed, *p<0.05, **p<0.01, ***p<0.001, ****p<0.0001 (n=4; two-way ANOVA with Tukey’s multiple comparison test).

**Table S5. ErbB4 isoform expression in human cingulate cortex and corpus callosum.**


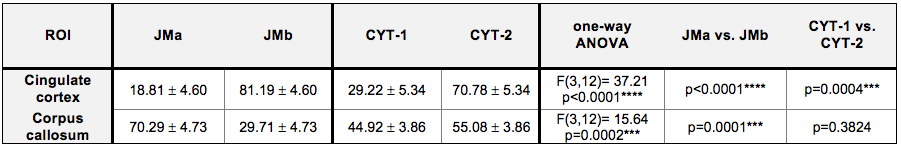


Relative abundance of ErbB4 isoforms in the human cingulate cortex and corpus callosum analyzed by TaqMan qRT-PCR. Values represent the mean ± SEM. Adjusted p values are listed, *p<0.05, **p<0.01, ***p<0.001, ****p<0.0001 (n=4; one-way ANOVA with Tukey’s multiple comparison test).

**References**

1. Tidcombe H, Jackson-Fisher A, Mathers K, Stern DF, Gassmann M, Golding JP (2003) Neural and mammary gland defects in ErbB4 knockout mice genetically rescued from embryonic lethality. Proc Natl Acad Sci U S A 100 (14):8281-8286. doi:10.1073/pnas.1436402100

2. Deng Y, Kim B, He X, Kim S, Lu C, Wang H, Cho SG, Hou Y, Li J, Zhao X, Lu QR (2014) Direct visualization of membrane architecture of myelinating cells in transgenic mice expressing membrane-anchored EGFP. Genesis 52 (4):341-349

3. Hughes EG, Kang SH, Fukaya M, Bergles DE (2013) Oligodendrocyte progenitors balance growth with self-repulsion to achieve homeostasis in the adult brain. Nat Neurosci 16 (6):668-676. doi:10.1038/nn.3390

4. Tamamaki N, Yanagawa Y, Tomioka R, Miyazaki J, Obata K, Kaneko T (2003) Green fluorescent protein expression and colocalization with calretinin, parvalbumin, and somatostatin in the GAD67-GFP knock-in mouse. J Comp Neurol 467 (1):60-79. doi:10.1002/cne.10905

5. Wang F, Flanagan J, Su N, Wang LC, Bui S, Nielson A, Wu X, Vo HT, Ma XJ, Luo Y (2012) RNAscope: a novel in situ RNA analysis platform for formalin-fixed, paraffin-embedded tissues. J Mol Diagn 14 (1):22-29. doi:10.1016/j.jmoldx.2011.08.002

6. Vullhorst D, Ahmad T, Karavanova I, Keating C, Buonanno A (2017) Structural Similarities between Neuregulin 1-3 Isoforms Determine Their Subcellular Distribution and Signaling Mode in Central Neurons. J Neurosci 37 (21):5232-5249. doi:10.1523/JNEUROSCI.2630-16.2017

7. Carpenter AE, Jones TR, Lamprecht MR, Clarke C, Kang IH, Friman O, Guertin DA, Chang JH, Lindquist RA, Moffat J, Golland P, Sabatini DM (2006) CellProfiler: image analysis software for identifying and quantifying cell phenotypes. Genome Biol 7 (10):R100. doi:10.1186/gb-2006-7-10-r100

8. Neddens J, Buonanno A (2010) Selective populations of hippocampal interneurons express ErbB4 and their number and distribution is altered in ErbB4 knockout mice. Hippocampus 20 (6):724-744. doi:10.1002/hipo.20675
